# COVID-19 Experiences, Behaviors, Beliefs, and Well-Being Among Students and Employees at a University In Rural Appalachia: A Cross-Sectional Study

**DOI:** 10.13023/jah.0304.09

**Published:** 2021-10-25

**Authors:** Lauren Wisnieski, Kimberly A. Carney, Jenny L. Thornley

**Affiliations:** College of Veterinary Medicine, Lincoln Memorial University, Harrogate TN

**Keywords:** Appalachia, mental health, COVID-19, social distancing, quarantine, adherence

## Abstract

**Introduction:**

In response to the coronavirus disease (COVID-19) pandemic, most universities experienced drastic operational changes with shifts to online learning, work-from-home policies, and social distancing measures. These changes have caused concern for social isolation and mental health.

**Purpose:**

This cross-sectional study explores differences in COVID-19 experiences, behaviors, beliefs, and well-being among students and employees (faculty and staff) at a rural Appalachian university.

**Methods:**

Data were collected with an online anonymous survey in September–October 2020 using convenience sampling. The survey measured multiple domains including COVID-19-related (1) beliefs, (2) symptoms and diagnoses, (3) exposure and preventive behavior, and (4) social, mental, and financial health. Chi-square tests and linear regression models were used to determine differences in survey responses between students and employees.

**Results:**

The final sample used for analysis included 416 respondents. The majority of respondents believed COVID-19 was a serious disease and followed mask and social distancing guidelines, although employees were more likely to adhere to mask and social distancing guidelines compared to students. Most of the respondents (>50%) reported feeling more stressed, anxious, and sad since the pandemic began. Students were more impacted by the pandemic compared to employees as measured by the mental, social, and financial impact scale. A limitation of this study was that convenience sampling was used instead of a probability sampling technique, which limits the inference that can be made from the results.

**Implications:**

There may be a need for greater mental health support among university employees and students. However, future studies should confirm these findings.

## INTRODUCTION

Universities have experienced major educational disruption and have undergone radical operational transformations in response to the coronavirus disease (COVID-19) pandemic, with shifts to online learning, greater reliance on digital technologies, and social distancing. The dramatic changes brought on by the COVID-19 pandemic have been shown to increase risk of negative mental health symptoms, such as depression and anxiety, in university students, faculty, and staff.^1^–^3^ Although studies have helped elucidate the mental health effects of the COVID-19 pandemic, few studies have focused on universities located in rural Appalachia.^1^ Rural Appalachia is a traditionally underserved population that is burdened with health and socioeconomic disparities, and with high rates of substance abuse and dependence.^4^ The rate of poverty in Appalachia subregions ranges from 13.6% (Northern Appalachia) to 23.5% (Central Appalachia) with an overall average of 15.2%, which is higher than the national average (13.4%).^5^ These disparities make this region especially sensitive to the financial, social, and mental health effects of the COVID-19 pandemic. There have been alarming reports of the detrimental effects of increased isolation in rural Appalachia, including higher incidence of relapse, overdoses, and deaths from substance-abuse disorders.^4^ In addition, students, faculty, and staff at universities in this region experience additional barriers due to disparities in internet access,^5^ which makes it difficult to connect with others and complete work or school assignments.

Understanding the differences in COVID-19 beliefs, experiences, and well-being between students, faculty, and staff can lead to better resource planning and allocation. For example, students may be in greater need of financial resources compared to staff or faculty due to lower socioeconomic status and financial instability.^6^ Studies in Italy and Spain have reported that students experience greater negative mental health effects caused by the pandemic, with higher prevalence of anxiety, stress, and depression compared to staff.^2^,^3^ However, this comparison has not been examined among students, faculty, and staff in Appalachia. To the best of our knowledge, no studies have examined differences in COVID-19 beliefs and adherence to safety guidelines between students, faculty, and staff.

Given the gaps in the literature outlined above, the objective of this study was to describe results from a cross-sectional survey conducted in September and October 2020 on COVID-19 experiences, behaviors, beliefs, and well-being in employees (faculty and staff) and students at a university based in rural Appalachia. At the time of the survey, most university activities were restricted to virtual platforms and faculty and staff were encouraged to work remotely.

## METHODS

The Strengthening the Reporting of Observational Studies in Epidemiology (STROBE) checklist for cross-sectional studies was followed for reporting of this study.^7^ This cross-sectional study was conducted September through October 2020 among faculty, staff, and students at a private university located in the central Appalachian region. Although the main campus was primarily targeted, respondents from off-site campuses located in multiple locations throughout the United States were also surveyed. The survey measured multiple domains, including (1) COVID-19 beliefs; (2) COVID-19 symptoms and diagnoses; (3) COVID-19 exposure and preventive behavior; (4) social, mental, and financial health; and (5) demographics. Questions were sourced through other previously published studies when possible.^8^–^16^ If relevant survey questions were not already available, questions were developed.

Questions developed by researchers included role, state, gender, age, income, marital status, whether they were tested for COVID-19, and whether they had COVID-19. All other questions were adapted from other sources. Survey questions are summarized in Table 1. Prior to sending out the survey invitation, a sample size calculation was performed to determine the number of respondents needed to report results with a certain level of precision. The parameters of the sample size calculation were: α = 0.05, a confidence level of 95%, and population size of 6276. In total, 363 respondents were needed. After Lincoln Memorial University Institutional Review Board approval (#941 V.1), the survey was sent through a Qualtrics survey link. Respondents were invited by direct solicitation through e-mail and announcements posted in buildings on campus. Respondents did not receive any compensation for completing the survey. Informed consent was obtained from all participants through electronic consent on the first question of the survey. If consent was confirmed, the survey continued to the next question.

To improve the accuracy and validity of the survey results, survey responses were removed if (1) the respondent failed to identify as a staff, student, or faculty member of the university; or (2) 50% or more of the questions were not answered. In addition, for the purpose of this analysis, respondents were dropped that reported that they were both faculty/staff and a student to allow for the comparison between these groups. To better target rural Appalachians, respondents were excluded that did not report what state they were in, that lived in non-Appalachian states, or lived in large cities. However, information was not collected to determine if they lived in Appalachian counties within those states.

Data were analyzed using Stata version 14.2 (College Station TX). Multiple scales were constructed based on the survey topics, including (1) belief of COVID-19 seriousness (α = 0.88); (2) COVID-19 preventive measure adherence (α = 0.80); (3) social, mental, and financial impact (α = 0.83); (4) social impact subscale (α = 0.92); and the (5) mental health impact subscale (α = 0.88) as described in Table 1. Scale reliability was assessed using Cronbach’s α. Scales were only used if they showed acceptable internal reliability (α ≥ 0.70). Categories of responses with a very small number of observations were combined for statistical purposes. Faculty and staff were combined into one category. In descriptive analyses, chi-square tests were used to determine if there were any significant differences in responses between employees and students for categorical variables. Fishers’ exact tests were used when expected counts were less than 5. Adjusted and unadjusted mixed effects linear regression models were built for the main outcomes of interest: (1) belief of COVID-19 seriousness score, (2) COVID-19 preventive measure adherence score, and (3) mental, social, and financial impact score and subscale scores. Adjusted analyses were adjusted for gender, age, income, and marital status. A random intercept for state was included in all models to adjust for shared variance at the state level. Normality of residuals was visually checked using histograms. Normality assumptions were not violated for any of the analyses. Statistical significance was set at P≤0.05.

## RESULTS

In total, 548 responded, which is approximately 8.7% of all students, staff, and faculty at the university and the university’s satellite campuses. After implementing the exclusion criteria (missing role [n = 3], both a student and faculty/staff [n = 11], missing responses to >50% of the questions [n = 3], missing information on what state they were in or they lived in non-Appalachian states [n = 87] or lived in a large city [n = 31]), the final sample included 416 respondents. Table 2 summarizes the demographic characteristics of the sample. Most of the respondents were female (73.5%) and white (91.8%). Due to the low racial and ethnic diversity of the sample and underlying population, race and ethnicity were grouped as white versus nonwhite for reporting purposes.

### COVID-19 experiences, behaviors, beliefs, and well-being

The most common symptoms participants reported having since the COVID-19 pandemic began were headache (34.4%), runny nose (24.3%), and fatigue (20.2%), although these symptoms cannot be attributed to having COVID-19. The percent of participants that were tested for COVID-19 at some point since the pandemic began was 38.9%. The test positivity rate was higher than the national average (9.4% versus 8.2%, respectively).^17^ When asked about their feelings about the state of the COVID-19 pandemic, 45.7% reported that they believed the worst was yet to come, 38.2% reported that they believed the worst is behind us, and 10.4% reported that they believed that COVID-19 is not and will not be a major problem (remaining 5.7% preferred not to say). Most respondents (76.7%) somewhat to strongly agreed that COVID-19 is a serious disease. When asked about restrictions set by the state they resided in for the majority of time since the pandemic began, 42.2% reported that they believed the restrictions were the right balance, 43.6% reported they were not restrictive enough, and 14.2% believed restrictions were too restrictive. The majority of the sample (71.4%) reported that they wore cloth face coverings at all times when in public, avoided gatherings of 10 or more people most of the time or always (70.3%), and kept 6 or more feet apart from others most of the time or always (64.7%).

In response to questions related to mental health, 23.3% and 27.4% reported that they lacked companionship and felt isolated from others, respectively, most of the time or always during the last 2 weeks. The majority of respondents reported that they at least somewhat to strongly agreed that they feel more stressed (80.2%), have more anxiety (74.1%), and feel sadder (56.7%) since the COVID-19 pandemic began.

The majority of respondents (52.4%) reported that that COVID-19 pandemic did not cause financial problems for them. However, 5.1% reported that the COVID-19 pandemic caused a great deal of financial problems for them.

### Differences between employees and students

There were many differences in COVID-19 experiences, behaviors, beliefs, and well-being between employees and students (see Tables 2 and 3). A higher prevalence of employees compared to students reported that they spent 3 hours or more on average outside their house during the last 2 weeks (88.1% versus 69.5%, respectively). The top source of COVID-19 information was less likely to be friends, family, or social media among employees compared to students (11.5% versus 30.9%, respectively).

Employees had significantly (P=0.03) higher belief of COVID-19 seriousness scores in adjusted analyses, but not in unadjusted analyses. Employees had significantly higher COVID-19 preventive measure adherence scores compared to students in unadjusted analyses, meaning they were more likely follow adherence guidelines, but not in adjusted analyses (Table 3). Students reported to be more impacted mentally, socially, and financially by COVID-19 (P<0.01) in both adjusted and unadjusted analyses (Table 3) and were more likely to have sought mental health treatment within the past 2 weeks compared to employees (P<0.01) (Table 2). Analysis of the mental, social, and financial impact subscales revealed that students reported being impacted more socially and financially compared to employees in both adjusted and unadjusted analyses (Table 3). However, students had significantly greater mental health impact scores compared to employees in only unadjusted analyses.

## DISCUSSION

This study examined if there were differences in COVID-19 experiences, behaviors, beliefs, and well-being among employees (faculty and staff) and students at a university based in rural Appalachia. The majority of respondents believed that COVID-19 is a serious disease and followed social distancing and mask guidelines almost all the time or always. To the best of our knowledge, this is the first study that reported on differences in COVID-19 social distancing and mask mandates adherence in university employees and students. Employees were more likely than students to adhere to guidelines in unadjusted analyses, but after adjustment for confounders, adherence scores were not significantly different between employees and students. Employees were older than students on average, therefore they may be more likely to adhere to guidelines due to higher risk of COVID-19 and complications.^18^ In fact, COVID-19 adherence scores increased with age, with lowest adherence in the lowest age group (18 to 24 years) and highest adherence scores in the highest age group (65 years and older; data not shown). Indeed, previous research has shown that younger adults are less adherent to social distancing guidelines compared to older adults.^19^ In addition, students may be less likely to self-isolate due to the higher risk of pandemic-related mental, social, and financial issues as demonstrated by this study. A previous study found that loneliness was associated with lower engagement in COVID-19 preventive behaviors.^20^ However, Wright and associates^21^ reported contrasting results and found that mental health, wellbeing, loneliness, and social isolation were not predictive of compliance. Additional research is needed to determine if mental health can affect adherence to preventive measures. Lastly, students may be less likely to adhere to mandates due to not believing COVID-19 is as serious as employees believe. In fact, student COVID-19 seriousness scores were lower than employees. Analyses of differences in seriousness scores between students and employees were borderline significant (P=0.055) in unadjusted analyses but were significant (P=0.03) in adjusted analyses.

In this study, a high prevalence of pandemic-related mental health symptoms were reported, with the majority of participants (>50%) believing that they feel more stressed, have more anxiety, and feel sad more often since the pandemic began. As mentioned above, students had significantly higher COVID-19 mental, social, and financial impact scores compared to employees, meaning they were more impacted by the pandemic on these factors. These findings are similar to previous studies that compared students and employees at universities in Spain^2^ and in Italy,^3^ which reported that students experienced greater effects of the pandemic on stress, anxiety, depression, and sleep. However, after adjustment of confounders, students did not have significantly different mental health impact subscale scores. This could be due to differences in mental health impact due to gender and age. Males had significantly lower mental health impact scores (data not shown) and there were more males in the employee group. Those that were 45 years old and older were also more likely to have lower mental health impact scores (data not shown) and were more likely to be employees than students. However, there are very few previous studies investigating the association of age and pandemic-related social isolation.^22^ Birditt and associates^22^ found that younger people were more likely to report higher degrees of pandemic-related social isolation and stress, although others have reported a high level of concern for social isolation among older adults.^23^ In the current study, it was also found that the students were more likely than employees to have sought mental health treatment in the past 2 weeks.

Although this study had a large sample size, there are limitations that must be addressed. The participants were not selected using a probability-based sampling technique, which increases the risk of selection bias. For instance, those affected more by the pandemic may be more likely to respond to the survey, which would inflate estimates of the impact of the pandemic on students and employees. In addition, convenience sampling limits what inferences can be made from the results, because the study population might not be representative of the underlying population (i.e., all employees and students at the university that are located in Appalachia). However, estimates of race and gender demographics are very similar at the university level and in our study population. Although statistics on the entire underlying population were not available, undergraduate demographic data from the National Center for Education Statistics^24^ estimated that 70.5% of undergraduates were women and 85% were white non-Hispanic at the university, which is close to the frequencies reported in our study population. In addition, this study was cross-sectional, and no data were collected from the respondents prior to the pandemic. This makes the results prone to recall bias, because respondents were asked to recall how stressed, anxiety, and sad they felt pre-pandemic. Longitudinal studies that employ random sampling and methods that optimize response rates are needed to confirm the findings from this study. Lastly, this study was conducted in a predominantly white, rural setting, at a private university. Due to the small scope of the study, the results likely cannot be generalized outside of this type of population.

## IMPLICATIONS

The results indicate that there may be a need for greater mental health support for employees and students in Appalachia during the pandemic. Students may be especially vulnerable to social isolation and financial stress, so if interventions are implemented, they should target this sensitive population during natural disasters, pandemics, and other events that disrupt educational activities. Examples of potential interventions at the university level include implementing policies that increase work–life balance, including courses in the curriculum that address the management of mental and financial health, requiring occupational health and safety training, altering assessment scales (i.e., scales with less categories are less stressful), offering counseling and stress-reduction interventions, and social marketing. However, studies examining the effect of mental health interventions at universities are scarce.^25^ Given the limited generalizability of the present study, future studies should be done to confirm the findings of the present study using a probabilistic sampling strategy and in additional universities throughout Appalachia. In addition, studies should be conducted to determine what interventions are most effective at supporting students and employees during times of educational disruption. Results from this study can be used to demonstrate the serious negative consequences of the pandemic and to encourage social distancing guidelines among students and employees. Universities should consider policies and communications that target students as they return to campus.

**SUMMARY BOX**

**What is already known on this topic?**

The COVID-19 pandemic drastically disrupted normal operations of universities across the country, causing concern for increased risk for mental health symptoms and social isolation among students and employees (faculty and staff). There are very few studies on the effect of the COVID-19 pandemic on students and employees at rural Appalachian universities.

**What is added by this report?**

The current study investigated differences in COVID-19 experiences, behaviors, beliefs, and well-being among students and employees at a rural Appalachian university. The results indicated that, in this particular sample, students are more vulnerable to the effects of social isolation and financial stress and are less likely to adhere to social distancing guidelines compared to employees.

**What are the implications for future research?**

Given the limited generalizability of the present study, future studies should confirm these findings in additional universities in Appalachia. Future research should involve developing and testing interventions that aim to support students during the COVID-19 pandemic, natural disasters, or other events that disrupt normal university activities.

## Figures and Tables

**Table 1:** Measures of COVID-19 experiences, behaviors, beliefs, and well-being

Predictor Item(s)	Question/Measure	Response Options/Predictor Variable
Role (1 item)	Are you faculty, student, or staff?	Faculty, Student, Staff (Check all that apply)
Gender (1 item)	What is your gender?	Female, Male, Other, Prefer not to say
Age (1 item)	What is your current age?	18 to 24, 25 to 34, 35 to 44, 45 to 54, 55 to 64, 65 and older
Income (1 item)	What is your household income from all sources before taxes?	<$20,000; $20,000 to <$30,000; $30,000 to <$40,000; $40,000 to <$50,000; $50,000 to <$60,000; $60,000 to <$70,000; $70,000 to $90,000; $90,000 to <$100,000; $100,000 or more
Marital status (1 item)	What is your current marital status?	Married, Divorced, Widowed, Separated, Never Married, Prefer not to say
Race (1 item)	What is your race or origin?	White, Black or African American, Asian, American Indian or Alaska Native, Native Hawaiian or Pacific Islander, Other, Prefer not to say (Check all that apply)
Hispanic (1 item)	Are you of Hispanic, Latino, or Spanish origin?	No- not of Hispanic, Latino, or Spanish origin, Yes- Mexican, Mexican American, Chicano, Yes-Puerto Rican, Yes-Cuban, Yes-another Hispanic, Latino, or Spanish, origin, Prefer not to say (Check all that apply)
State (1 item)	Since the COVID-19 pandemic began, what U.S. state did you primarily spend your time in?	Drop down of all states, plus an option for N/A- out of country or prefer not to say, combined into regions
Neighborhood type (1 item)	Since the COVID-19 pandemic began, what type of community did you live in?	Rural area, large city, suburb near a large city, small city or town
Information source (1 item)	Where do you get most of your information about COVID-19?	Ranked the top four news sources (broadcast TV, cable TV, etc) in the order used most often (1=most frequently, 2=2nd most frequently, 3=3rd most frequently, 4=4th most frequently)
Mental health treatment (1 item)	Have you sought mental health treatment in the past 2 weeks?	Yes/No
Time spent outside (1 item)	In the last 2 weeks, on average, about how much time daily did you spend outside of your household?	No time, <30 minutes, 30 minutes to 1 hour, 1 hour to 2 hours, 2 hours to 3 hours, 3 hours or more
Tested for COVID-19 (1 item)	Have you been tested for COVID-19?	Yes, No, Not sure, or Prefer not to say
Had COVID-19 (2 items)	Whether the respondent either tested positive or was told that they had COVID-19 by a provider	Respondent was categorized as having had COVID-19 if they answered yes to either question.
Belief of COVID-19 seriousness (6 items)	The extent to which the respondent felt that the COVID-19 outbreak was a major problem, that the guidelines set by the state should be stricter, and that businesses and non-essential medical care operations should be reduced	COVID-19 seriousness score*† created from multiple items. Final scale ranges from 1 (not serious) to 7 (serious).
COVID-19 symptoms (19 items)	Which of the following symptoms have you experienced since the COVID-19 outbreak began in your area?	The number of symptoms experienced was summed and then categorized as 1, 2, and 3 or more COVID-19 symptoms.
COVID-19 exposure (3 items)	The extent to which the respondent spent time outside their household and spent time with someone who had COVID-19	The sum of the number of people they had contact with in and outside their household that had COVID-19, categorized to 0, 1 and 2 or more
COVID-19 preventive measure adherence (3 items)	The extent to which the respondent wore cloth face coverings in public, avoided large groups, and kept 6 or more feet apart from others	COVID-19 adherence score*,§ ranging from 1 (never) to 5 (always).
Social, mental, and financial impact (6 items)	The extent to which the respondent felt lonely or isolated in the past 2 weeks, experienced more stress, anxiety, and sadness since COVID-19 outbreak began, and experienced financial problems because of COVID-19	Social, mental, and financial impact score*,¶ ranging from 1 (highly affected) to 5 (not affected at all). Social impact** (2 items), mental health impact†† (2 items), and financial impact (1 item) sub-scales also evaluated

* Continuous variable.

† Responses were standardized to a 7-point Likert scale and responses were averaged to create score. Cronbach’s α = 0.88.

§ Responses were measured on a 5-point Likert scale and responses were averaged to create score. Cronbach’s α = 0.80.

¶ Responses were standardized to a 5-point Likert scale and responses were averaged to create score. Cronbach’s α = 0.83.

** Responses were standardized to a 5-point Likert scale and responses were averaged to create score. Cronbach’s α = 0.92.

†† Responses were standardized to a 5-point Likert scale and responses were averaged to create score. Cronbach’s α = 0.88.

**Table 2:** Demographic information and COVID-19 experiences, behaviors, beliefs, and well-being among employees and students (n = 416)*

	Employees (n = 177)	Students (n = 239)	Row total (n = 416)	Chi-square (df)	P-value
**Gender (n[%])** †					
Male	53 (31.0)	55 (23.3)	108 (26.5)	3.1 (1)	0.08
Female	118 (69.0)	182 (76.8)	300 (73.5)	–	
**Age (years) (n[%])**				226.5 (1)	<0.01
18 to 24	8 (4.6)	154 (64.4)	162 (39.0)	–	–
25 to 34	33 (18.8)	65 (27.2)	98 (23.6)	–	–
35 to 44	43 (24.4)	10 (4.2)	53 (12.8)	–	–
45 to 54	44 (25.0)	9 (3.0)	53 (12.8)	–	–
55 to 64§	–	–	–	–	-
65 and older§	–	–	–	–	–
**White (versus nonwhite) (n[%])**	164 (92.7)	218 (91.2)	382 (91.8)	0.28	0.60
**Income (in thousands) (n[%])**				81.4 (8)	<0.01
<20§	–	–	–	–	–
20 to <30§	–	–	–	–	–
30 to <40	12 (7.2)	20 (8.5)	20 (8.5)	–	–
40 to <50	11 (6.6)	12 (5.1)	12 (5.1)	–	–
50 to <60	13 (7.8)	15 (6.4)	15 (6.4)	–	–
60 to <70	18 (10.8)	16 (6.8)	16 (6.8)	–	–
70 to <80	18 (10.8)	18 (7.6)	18 (7.6)	–	–
80 to <100	13 (7.8)	9 (3.8)	9 (3.8)	–	–
100 and greater	71 (42.8)	43 (18.2)	43 (18.2)	–	–
**Marital status (n[%])**				182.2 (2)	<0.01
Married	130 (74.7)	35 (14.6)	165 (40.0)	–	
Never married	25 (14.4)	194 (81.2)	219 (53.0)	–	
Divorced/Widowed/Separated	19 (10.9)	10 (4.2)	29 (7.0)	–	
**Region (n[%])**				5.5 (2)	0.06
Midwest	14 (7.9)	33 (13.8)	47 (11.3)	–	–
Northeast	10 (5.7)	21 (8.8)	31 (7.5)	–	–
Southeast	153 (86.4)	185 (77.4)	338 (81.3)	–	–
**Neighborhood type (n[%])**				16.0 (2)	<0.01
Rural area	109 (61.6)	101 (42.3)	210 (50.5)	–	–
Small city or town	43 (24.3)	78 (32.6)	121 (29.1)	–	–
Suburb near a large city	25 (14.1)	60 (25.1)	85 (20.4)	–	–
**Top source of COVID-19 information (n[%])**				24.4 (3)	<0.01
TV	33 (19.0)	21 (9.6)	54 (13.7)	–	–
Federal, state, or local government officials	77 (44.3)	80 (36.4)	157 (39.9)	–	–
Friends, family, or social media	20 (11.5)	68 (30.9)	88 (22.3)	–	–
Online news sites, radio news, or print news	44 (25.3)	51 (23.2)	95 (24.1)	–	–
**Time spent outside home daily on average during the last 2 weeks (n[%])**					
No time§	–	–	–	24.0 (5)	<0.01¶
<30 minutes§	–	–	–	–	–
30 minutes to <1 hour	9 (5.1)	15 (6.3)	24 (5.8)	–	–
1 hour to <2 hours§	–	–	–	–	–
2 hours to <3 hours	7 (4.0)	28 (11.7)	35 (8.4)	–	–
3 hours or more	155 (88.1)	166 (69.5)	321 (77.4)	–	–
**Tested for COVID-19 (n[%])** **	61 (34.5)	101 (42.3)	162 (38.9)	2.6 (1)	0.11
**Test positivity rate for COVID-19 (n[%])**	3 (5.0)	12 (12.1)	15 (9.4)	2.2 (1)	0.14
**Sought mental health treatment in past 2 weeks (n[%])**	7 (4.1)	37 (16.2)	44 (11.1)	14.5 (1)	<0.01
**Number of COVID-19 symptoms experienced (n[%])**					
0	87 (49.2)	129 (54.0)	216 (51.9)	3.5 (3)	0.32
1	17 (9.6)	24 (10.0)	41 (9.9)	–	–
2	20 (11.3)	15 (6.3)	35 (8.4)	–	–
3 or more	53 (29.9)	71 (29.7)	124 (29.8)	–	–
**Number of people with COVID-19 survey respondent was exposed to (n[%])**					
0	149 (84.2)	195 (81.6)	344 (82.7)	2.3 (2)	0.31
1	21 (11.9)	26 (10.9)	47 (11.3)	–	–
2 or more	7 (4.0)	18 (7.5)	25 (6.0)	–	–

* Missing data: Information source (n = 22); sought mental health treatment (n = 18); income (n = 14); test positivity rate for COVID-19 (n = 3); time spent outside (n = 1).

† Analysis did not include those that responded “other” and “not reported.”

§ Data not released due to small sample size (cell sizes <5).

¶ Fisher’s exact.

** Analysis did not include those who responded “not sure.”

**Table 3:** Unadjusted and adjusted estimates from linear regression models comparing the belief of COVID-19 seriousness, COVID-19 preventive measure adherence, and mental, social, and financial health between students and employees (n = 416)*

	Unadjusted estimates for students (ref = employees)		Adjusted estimates† for students (ref = employees)	
Outcome	β (SE)	P-value	β (SE)	P-value
**Belief of COVID-19 seriousness score**	−0.29 (0.15)	0.055	−0.51 (0.24)	0.03
**COVID-19 preventive measure adherence score**	−0.36 (0.09)	<0.001	−0.17 (0.15)	0.27
**Mental, social, and financial impact score**	0.68 (0.08)	<0.001	0.37 (0.13)	0.004
*Mental health impact subscale*	0.46 (0.10)	<0.001	0.14 (0.16)	0.39
*Social impact subscale*	0.93 (0.12)	<0.001	0.62 (0.19)	0.001
*Financial impact subscale*	0.79 (0.12)	<0.001	0.54 (0.18)	0.003

* Missing data for items within scales: state restrictions (n = 1), COVID-19 belief (if worst is yet to come or worst is behind us) (n = 99), avoidance of gatherings of 10 or more people (n = 2), whether the respondent felt more stress (n = 1).

† Adjusted for gender, age, income, and marital status.
